# Patterns of Antibiotic Dispensing Among New Zealand Children: A Linkage Study

**DOI:** 10.1002/pds.70450

**Published:** 2026-08-13

**Authors:** Sharan Ram, Marine Corbin, Amanda Kvalsvig, Michael G. Baker, Andrea ‘t Mannetje, Amanda Eng, Jeroen Douwes

**Affiliations:** ^1^ Center for Public Health Research Massey University Wellington New Zealand; ^2^ Department of Public Health University of Otago Wellington New Zealand

## Abstract

**Background:**

Overuse of antibiotics contributes to antibiotic resistance, and may result in gut microbiome dysbiosis, potentially increasing the risk of chronic childhood conditions.

**Objectives:**

To examine patterns of antibiotic dispensing in utero and during the first 5 years of life.

**Methods:**

We conducted a retrospective cohort study of all children (*n* = 315 786) born in New Zealand from 2005 to 2010 using linked pharmaceutical dispensing data to ascertain antibiotic use during the mother's pregnancy and for the first 5 years of life. Descriptive analyses were conducted as well as negative binomial regression controlled for potential confounders.

**Results:**

In total, 96% of children had been dispensed ≥ 1 course of antibiotics by age five; for pregnant women this was 30%. Penicillin, particularly Amoxicillin, was most frequently dispensed during both periods. Postnatal antibiotic dispensing was higher for males (adjusted IRR 1.09, 95% CI 1.08–1.10); Māori (1.16, 1.15–1.17), Pacific peoples (1.32, 1.30–1.33), and Middle East Latin American and African (MELAA) ethnicities (1.07, 1.04–1.10); children in the highest deprivation quintile (1.16, 1.15–1.17) and those born pre‐term (1.05, 1.04–1.05); and urban children (1.21, 1.20–1.22). Low and high birthweight and caesarean deliveries were also associated with higher antibiotic dispensing. Similar patterns were observed for antibiotic dispensing for mothers during pregnancy. Dispensing rates were highest during winter months.

**Conclusion:**

Antibiotic dispensing is high in New Zealand children and pregnant women, with significant ethnic and socioeconomic differences, suggesting that current antibiotic stewardship programmes are not fully effective.

## Key Points

- Antibiotic use in early life is common and has been linked to antimicrobial resistance and potential long‐term adverse health outcomes, but nationally representative data covering both pregnancy and early childhood are limited.
- Nearly all New Zealand children (96%) had been dispensed antibiotics by age five, and 30% of mothers received antibiotics during pregnancy, with substantial ethnic and socioeconomic inequities. Dispensing was highest among Māori and Pacific peoples, more deprived populations, and during winter months.
- Persistently high antibiotic dispensing suggests there is a need for more effective antimicrobial stewardship, especially in infants and young children.

## Plain Language Summary

Antibiotics are important for the management of bacterial infections. However, frequent and widespread use can increase antibiotic resistance and may affect long‐term health through changes in gut bacterial composition and associated effects on immune system development. This national study examined antibiotic dispensing during pregnancy and the first 5 years of life for over 315 000 children born in New Zealand. Almost all children (96%) had been dispensed at least one course of antibiotics by age five, and nearly one in three mothers received antibiotics while pregnant. Antibiotic dispensing was higher among Māori (the indigenous population of New Zealand) and Pacific children (with Pacific Island nations ancestry), children living in more deprived areas, boys, children born pre‐term or by caesarean section, and those living in urban areas. Most antibiotics were dispensed in winter, with the penicillin‐based medicines most commonly dispensed. Similar patterns were observed for antibiotic use during pregnancy. These findings show that antibiotic use is very high in New Zealand and is unevenly distributed across different population groups. This suggests that overprescribing of antibiotics may be widespread indicating that more targeted approaches may be needed to help prevent further antibiotic resistance.

**Abbreviations**
ATC: Anatomical Therapeutic Chemical
IDI: integrated data infrastructure
NZ: New Zealand

## 1 Introduction

Antibiotics are the most common type of medication prescribed. However, overuse of antibiotics is increasingly common, thus contributing to antimicrobial resistance (AMR) [1, 2], which has been estimated to be a leading cause of death globally [3]. It may also, particularly in early life, result in gut microbiome dysbiosis [4], which has been suggested to increase the risk of several chronic conditions [5, 6], including Type 1 diabetes [7], attention deficit hyperactivity disorder [8, 9], inflammatory bowel disease [10], and asthma [4, 11]. However, the causal mechanisms remain an area of active investigation.

Global antibiotic consumption increased by 46% from 2000 to 2018 (from 9.8 to 14.3 defined daily doses (DDD)/1000 people/day), with relatively stable rates in high‐income countries but a steep rise in low‐ and middle‐income countries (LMICs), particularly North Africa, the Middle East (111%), and South Asia (116%) [12]. Children < 5 years consume antibiotics at rates approximately three times that of adults [13], and antibiotics sales for use in children from 70 middle‐ and high‐income countries showed that prescribing patterns for several countries were inconsistent with the World Health Organization's recommendation on antimicrobial stewardship (AMS). In particular, in 17 countries, notably China and India, > 20% of antibiotics consumed by children were broad‐spectrum, which carries a higher risk of antibiotic resistance, and overall use of key first‐line antibiotics was lower than recommended [14].

In New Zealand, community antibiotic consumption is among the highest of OECD countries; in 2013, it was higher than 22 of 29 European nations [15]. Furthermore, antibiotic use in childhood is widespread, with one study finding it was nearly universal by age 5 years [16]. To accommodate population growth and to address a shortage of medical prescribers, New Zealand introduced (in 2002) standing orders that allow for non‐physician healthcare professionals, such as dentists, nurses and pharmacists, to prescribe antibiotics [17, 18] potentially further increasing antibiotic consumption.

Policies to increase awareness of AMS and reduce the risk of AMR have been introduced in many countries [19, 20] but these have often not been effective [21]. For example, in New Zealand, a recent evaluation of education aimed at parents about the ineffectiveness of antibiotics for viral infections and its potential to cause adverse health effects successfully lowered parent expectations about receiving antibiotics; however, it did not affect the prescribing behaviour of general practitioners [22].

Global and local variations in antibiotic use are likely due to a combination of underlying differences in the incidence of bacterial infections and differences in prescribing practices, healthcare policies, socio‐demographic factors, and consumer expectations; however, the specific drivers are not fully understood. The current study assessed the patterns of antibiotic dispensing from the prenatal period and during the first 5 years of life in a national cohort of New Zealand children, whilst considering differences in ethnicity, sex, rurality, and other demographic, perinatal, and maternal factors.

## 2 Design and Methods

### 2.1 Study Population

This retrospective linkage study included all children (*n* = 315 786) born in New Zealand between October 2005 and December 2010. Patterns of antibiotic dispensing during the first 5 years of life and for their mothers during pregnancy were analysed. This study was nested within a larger study of early‐life exposures and chronic childhood conditions [23], which required sufficient follow‐up time for disease development, hence focusing on births in the 2005–2010 period. Children who died before age five were excluded from the analysis.

The study was conducted using the Statistics New Zealand (Stats NZ) Integrated Data Infrastructure (IDI), a longitudinal meta‐dataset linked at the individual level consisting of de‐identified data from government agencies (e.g., health, social, economic), Stats NZ surveys (including the 2018 census) and non‐government organisations [24]. Data for this study were accessed for research purposes on 15 February 2023.

### 2.2 Antibiotic Dispensing

Antibiotic dispensing during pregnancy and from 0 to 5 years was identified using pharmaceutical data from community pharmacies. In New Zealand, antibiotics for systemic use are only available with a prescription and recorded in the Pharmaceutical Collection [25]. This does not include information on hospital inpatient dispensing or medications provided directly by a medical practitioner in an emergency [26]; this information is therefore not available.

The primary outcome was the total number of systemic antibiotic courses dispensed per child during pregnancy and from 0 to 5 years. Prescription dispensing dates and the number of purchases were identified, with each prescription categorised by class according to the Anatomical Therapeutic Chemical (ATC) classification J01 ‘Antibiotics for systemic use’ (e.g., penicillin's, cephalosporins, sulphonamides). Dispensing dates were used to count the number of antibiotic prescriptions per child for each year of life or each trimester of pregnancy.

### 2.3 Other Variables

Antibiotic dispensing was compared across demographic characteristics, including sex, ethnicity, rurality, and socioeconomic position as defined by the 2018 NZ Deprivation Index (NZDep; a census‐based index with a relative deprivation score assigned to each geographical meshblock of residence [27]). We also compared antibiotic dispensing for groups with different birthweights, modes of delivery, birth terms and maternal age at birth. We used prioritised ethnicity (in case of multiple ethnicities), which involved each person being allocated to a single ethnic group, using the following prioritised order: Māori, Pacific, Asian, Middle Eastern, Latin American & African (MELAA), Others, and NZ Europeans [28]. The Geographic Classification for Health (GCH) was used for urban–rural classification [29].

### 2.4 Statistical Analysis

SAS version 9.4 (SAS Institute, Cary, NC, USA) was used. The main antibiotic outcome was presented as the percentage of children who had been dispensed at least one course of antibiotics. We also presented cumulative percentages for different time periods.

To assess differences in antibiotic dispensing (expressed as the number of prescriptions in the five‐year follow‐up period) between different population groups and population characteristics we conducted negative binomial regression for both the prenatal and postnatal periods. We present both crude and adjusted incidence rate ratios (IRRs, with 95% confidence intervals (CIs)), with analyses adjusted for sex, ethnicity, NZDep, rurality, mode of delivery, birth term, birthweight, and maternal age at birth.

We assessed seasonal patterns of antibiotic dispensing in a subset (*n* = 8409) born in the last quarter of 2005. This allowed any recurring seasonal trends in antibiotic dispensing to be assessed over the full 5 years of follow‐up, with data presented as the percentage of children who were dispensed a course of antibiotics by calendar month and year. Seasons in New Zealand were defined as follows: summer, December–February; autumn, March–May; winter, June–August; and spring, September–November, aligning with the meteorological seasons in the Southern Hemisphere.

In compliance with the Stats NZ IDI confidentiality requirements, all frequencies were rounded to the nearest multiple of three, and percentages were calculated from the rounded counts (hence the number of total participants varying slightly, and percentages not adding up to exactly 100%). All statistical tests were performed on unrounded counts. All counts under six were suppressed and marked as ‘S’ in the tables.

### 2.5 Ethics

This study was approved by the Health and Disability Ethics Committees (20/CEN/7) and the Human Research Ethics Committee of the University of Otago (HD21/053). Microdata access approval was provided by Stats NZ.

## 3 Results

### 3.1 Study Population

Demographic characteristics of the cohort are detailed in Table 1.

**TABLE 1:** Demographic and other characteristics.

Variables	*N* (%)
Total	315 786
Sex	
Male	162 564 (51.5)
Female	153 219 (48.5)
Ethnicity	
NZ Europeans	144 306 (45.7)
Māori	98 547 (31.2)
Pacific	34 017 (10.7)
Asian	30 543 (9.7)
Middle Eastern, Latin American, and African (MELAA)	3819 (1.2)
Others	4560 (1.4)
Deprivation Index in quintiles	
1 (Lowest deprivation)	39 894 (13.2)
2	49 176 (16.3)
3	55 977 (18.6)
4	64 854 (21.5)
5 (Highest deprivation)	91 317 (30.3)
Rurality	
Urban	245 643 (83.8)
Rural	47 568 (16.2)
Birthweight	
Normal birthweight (NBW) [2500–4000 g]	247 785 (78.7)
High birthweight (HBW) [> 4000 g]	48 675 (15.5)
Low birthweight (LBW) [< 2500 g]	182 49 (5.8)
Type of delivery	
Non‐Caesarean Section	230 118 (73.0)
Caesarean Section (Elective)	33 051 (10.5)
Caesarean Section (Emergency)	42 642 (13.5)
Birth term	
Full‐term (≥ 39 weeks and ≤ 40 weeks)	167 568 (53.1)
Pre‐ & early term (≤ 38 weeks)	79 932 (25.3)
Late‐term (= 41 weeks)	55 692 (17.6)
Post‐term (≥ 42 weeks)	12 444 (3.9)
Maternal age at delivery (years)	
< 25	75 069 (23.8)
≥ 25 and < 30	74 421 (23.6)
≥ 30 and < 35	89 553 (28.4)
≥ 35	76 737 (24.3)

**FIGURE 1:**
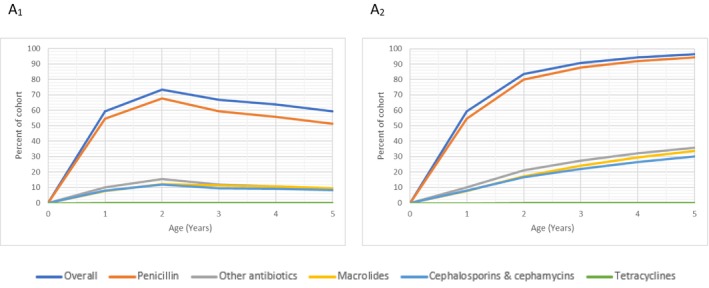
Percentage of children who were dispensed ≥ 1 antibiotic courses in each year of life from birth to 5 years by class of antibiotics. Figure 1A_1_ represents non‐cumulative data and Figure 1A_2_ represents cumulative data.

### 3.2 Postnatal Antibiotic Dispensing

For the whole study population, a total of 2 800 233 systemic antibiotic prescriptions were dispensed during the first 5 years. Penicillin was the predominant class (73.6%) and within this class, amoxicillin was dispensed the most (33.9%). Other antibiotic classes were dispensed as follows: 9.6% other, miscellaneous, 8.5% cephalosporins and cephamycins, and 8.4% macrolides. Tetracyclines accounted for < 1%.

We observed a steady increase in the proportion of children who were dispensed antibiotics for each month in the first year of life starting from 2.3% in the first month and peaking at 18.4% by 12 months. There was a gradual decline for the subsequent 48 months (Figure S1 & Figure 1A_1_). By the age of five, 96.0% had been dispensed at least one course of antibiotics; for years 1–4, the cumulative percentages were 59.1%, 83.4%, 90.4%, and 94.1%, respectively (Figure 1A_2_). On average, 64.4% of children were dispensed at least one antibiotic each year (year 1: 59.1%, year 2: 73.1%, year 3: 66.8%, year 4: 63.6%, and year 5: 59.3%).

**FIGURE 2:**
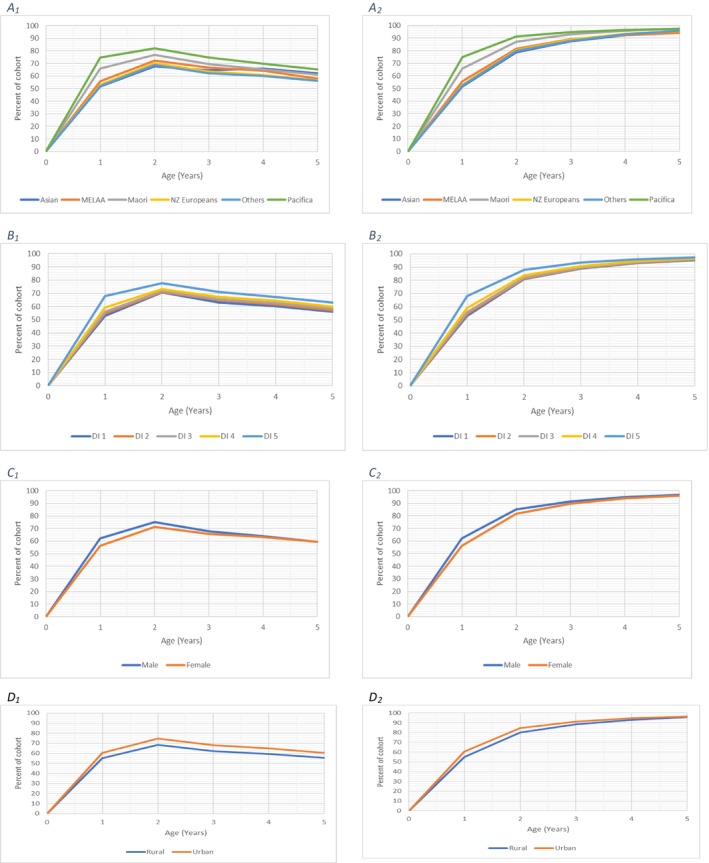
Percentage of children who were dispensed ≥ 1 antibiotic courses in each year from birth to age five by ethnicity (2A_1_ and 2A_2_), deprivation index (DI, 2B_1_ and 2B_2_) categorised by quintiles (DI1 is the least deprived and DI5 is the most deprived), sex (2C_1_ and 2C_2_), and rurality (2D_1_ and 3D_2_). Figures 2A_1_‐2D_1_ represent non‐cumulative data and Figures 2A_2_‐2D_2_ represent cumulative data.

Seasonal variation in antibiotic dispensing was evident, with elevated rates observed during winter and lower dispensing in summer (Figure S2).

#### 3.2.1 Differences in Antibiotic Dispensing Among Sub‐Groups

Antibiotic prescriptions (defined as having been dispensed at least one course of antibiotics) were higher in Pacific, Māori, and MELAA children compared to NZ European and Other ethnicities across each year (Figures 2A_1_ and 2A_2_). Dispensing in Asian children was similar to those of NZ European and Other children for the first 2 years, but it was higher for subsequent years (Figure 2A_1_). Following from this, Pacific, Māori and MELAA children also received their first antibiotics courses at younger ages, with mean ages ranging from 1.2, 1.4, and 1.4 years, respectively, as opposed to 1.5, 1.6 and 1.6 years for children of NZ European, Asian, and Other ethnicities, respectively.

The proportion of children dispensed ≥ 1 antibiotic course was higher for the most deprived compared to the least deprived (Figures 2B_1_ and 2B_2_). For the first 2 years boys were more likely to have been dispensed ≥ 1 course of antibiotics compared to girls, but in subsequent years this difference reduced with no difference observed in year five (Figures 2C_1_ and 2C_2_). The proportion of children in urban areas who were dispensed ≥ 1 course of antibiotics was higher than that of their rural counterparts (Figure 2D_1_). There was little or no difference in the proportion of children dispensed ≥ 1 course of antibiotics with different birthweights or for mode of delivery (Figure S3A,B).

#### 3.2.2 Number of Prescriptions and Incident Rate Ratios (IRRs) of Antibiotic Dispensing

**FIGURE 3:**
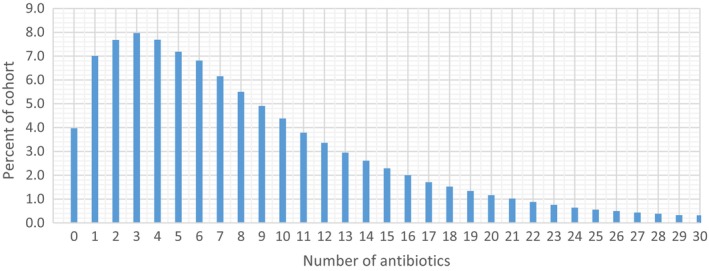
Distribution of the number of antibiotic courses dispensed per child during the first 5 years.

The distribution of the number of antibiotics dispensed per child by age five is shown in Figure 3. By age five, the median number of antibiotics dispensed was seven (IQR 3–12), with a further breakdown of median numbers by subgroups provided in Table 2.

**TABLE 2:** Median with interquartile range (IQR) of number of antibiotics dispensed in the first 5 years and incidence rate ratios (IRR) comparing different groups.

Explanatory variable	Median (IQR)	Crude IRR (95% CI)	*p*	Adjusted^a^ IRR (95% CI)	*p*
Gender					
Female	6 (3–12)	Ref.	Ref.	Ref.	Ref.
Male	7 (4–13)	1.09 (1.09–1.10)	< 0.0001	1.09 (1.08–1.10)	< 0.0001
Ethnicity					
NZ Europeans	6 (3–11)	Ref.	Ref.	Ref.	Ref.
Asian	7 (3–12)	1.07 (1.06–1.08)	< 0.0001	1.02 (1.01–1.03)	< 0.0001
MELAA	7 (3–12)	1.13 (1.10–1.16)	< 0.0001	1.07 (1.04–1.10)	< 0.0001
Māori	7 (4–13)	1.20 (1.19–1.21)	< 0.0001	1.16 (1.15–1.17)	< 0.0001
Others	6 (3–10)	0.96 (0.94–0.99)	0.0018	0.96 (0.94–0.99)	0.0024
Pacific	9 (5–15)	1.43 (1.41–1.44)	< 0.0001	1.32 (1.30–1.33)	0.0063
NZ Deprivation Index in quintiles					
1 (Lowest deprivation)	6 (3–11)	Ref.	Ref.	Ref.	Ref.
2	6 (3–11)	1.03 (1.02–1.04)	< 0.0001	1.03 (1.02–1.04)	< 0.0001
3	6 (3–11)	1.05 (1.04–1.06)	< 0.0001	1.04 (1.03–1.06)	< 0.0001
4	7 (3–12)	1.11 (1.10–1.12)	< 0.0001	1.08 (1.06–1.09)	< 0.0001
5 (Highest deprivation)	8 (4–14)	1.26 (1.25–1.27)	< 0.0001	1.16 (1.15–1.17)	< 0.0001
Rurality					
Rural	6 (3–10)	Ref.	Ref.	Ref.	Ref.
Urban	7 (4–13)	1.23 (1.22–1.24)	< 0.0001	1.21 (1.20–1.22)	< 0.0001
Mode of delivery					
Non‐Caesarean section	7 (3–12)	Ref.	Ref.	Ref.	Ref.
Caesarean section (elective)	7 (3–12)	1.04 (1.03–1.05)	< 0.0001	1.07 (1.06–1.09)	< 0.0001
Caesarean section (emergency)	7 (4–13)	1.05 (1.04–1.06)	< 0.0001	1.06 (1.05–1.07)	< 0.0001
Birth term (in weeks)					
Full‐term (≥ 39 and ≤ 40)	7 (3–12)	Ref.	Ref.	Ref.	Ref.
Pre‐ & early‐term (≤ 38)	7 (3–13)	1.05 (1.04–1.06)	< 0.0001	1.05 (1.04–1.05)	< 0.0001
Late‐term (= 41)	7 (3–12)	0.97 (0.96–0.98)	< 0.0001	0.98 (0.97–0.99)	< 0.0001
Post‐term (≥ 42)	7 (3–12)	0.99 (0.97–1.00)	< 0.0001	0.97 (0.95–0.98)	< 0.0001
Birthweight					
NBW (2500–4000 g)	7 (3–12)	Ref.	Ref.	Ref.	Ref.
HBW (> 4000 g)	7 (3–12)	1.03 (1.03–1.04)	< 0.0001	1.03 (1.02–1.04)	< 0.0001
LBW(< 2500 g)	7 (4–13)	1.07 (1.05–1.08)	< 0.0001	1.02 (1.00–1.03)	0.0101
Maternal age at birth					
< 25	7 (4–12)	Ref.	Ref.	Ref.	Ref.
≥ 25 and < 30	7 (3–12)	0.99 (0.98–1.00)	0.0793	1.03 (1.03–1.04)	< 0.0001
≥ 30 and < 35	7 (3–12)	0.95 (0.94–0.96)	< 0.0001	1.02 (1.01–1.03)	< 0.0001
≥ 35	6 (3–12)	0.93 (0.92–0.94)	< 0.0001	0.99 (0.99–1.01)	0.43

^a^ Adjusted for sex, ethnicity, deprivation index, rurality, mode of delivery, birth term, birthweight, and maternal age at birth.

The number of antibiotics courses dispensed was 1.7 prescriptions/child/year (SD 1.5). The number of antibiotics dispensed was higher for Māori, Pacific people, and MELAA, but also Asians (Table 2). It was also higher in more deprived, male, and urban children, and for those delivered by both elective and emergency caesarean. Children born preterm were dispensed more antibiotics courses compared to those born full‐term; children born post‐term and late‐term had lower dispensing rates. Dispensing rates were also higher for children with low as well as high birthweights compared to normal birthweight; some differences were also observed for maternal age at delivery (Table 2).

### 3.3 Antibiotic Dispensing in Mothers

**FIGURE 4:**
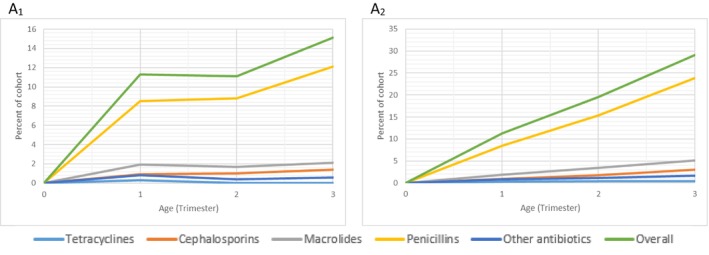
Percentage of mothers who were dispensed ≥ 1 antibiotic courses in each trimester from conception until delivery by class of antibiotics. Figure 4A_1_ represents non‐cumulative data and Figure 4A_2_ represents cumulative data.

Overall, 29.1% of mothers had been dispensed ≥ 1 course of antibiotics during pregnancy, with 11.3% having been dispensed antibiotics during the first, 11.1% during the second, and 15.1% during the third trimesters (Figure 4). The most frequently dispensed class of antibiotics was penicillin (73.7%), with sub‐classes amoxicillin and amoxicillin with clavulanic acid accounting for 62.2% of the penicillin class dispensed.

**FIGURE 5:**
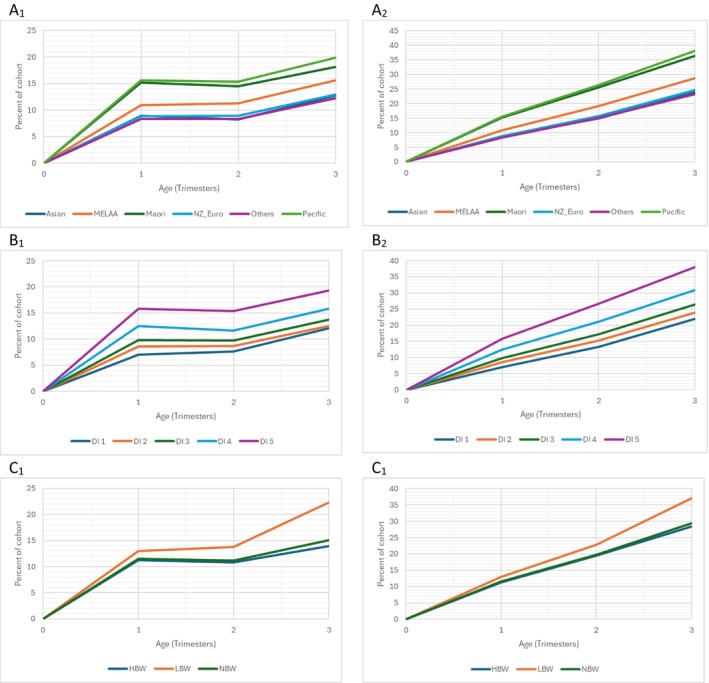
Percentage of mothers who were dispensed ≥ 1 antibiotic courses in each trimester of pregnancy from conception to birth by ethnicity (5A_1_ and 5A_2_), deprivation index (5B_1_ and 5B_2_) in quintiles (DI 1 is least deprived and DI 5 is the most deprived), and birthweight (5C_1_ and 5C_2_; Low (LBW) = [< 2500 g]; Normal (NBW) = [2500–4000 g]; High (HBW) = [> 4000 g]). Figures 5A1–5C1 represent non‐cumulative data and Figures 5A2–5C2 represent cumulative data.

#### 3.3.1 Differences in Antibiotic Dispensing Among Sub‐Groups

The proportion of mothers who were dispensed ≥ 1 course of antibiotics during pregnancy was considerably greater for Pacific, Māori, and MELAA mothers during each trimester of pregnancy (Figures 5A_1_ and 5A_2_). In particular, throughout the pregnancy, 38.7% of Pacific, 35.7% of Māori, and 31.6% of MELAA mothers were dispensed one or more courses of antibiotics, compared to 24.5%, 24.1% and 25.1% of NZ European, Asian and Other mothers, respectively.

The proportion of mothers dispensed ≥ 1 course of antibiotics in the most deprived category was nearly twice (39.5%) that of those in the least deprived category (21.6%) (Figure 5B_1_). The percentage of mothers dispensed ≥ 1 course of antibiotics was approximately one‐third higher among mothers who delivered children with low birthweights compared to mothers who delivered children with normal and high birthweights (Figures 5C_1_ and 5C_2_). The sex of the child was not associated with maternal antibiotic dispensing (Figure S4A). Similarly, there was no difference in the proportion of mothers dispensed ≥ 1 course of antibiotics between urban and rural mothers (Figure S4B).

#### 3.3.2 Number of Antibiotics Dispensed and Incident Rate Ratios (IRRs)

In total, 30% of mothers had been dispensed antibiotics during pregnancy (Figure 4A_2_). The incidence of antibiotic dispensing for mothers pregnant with boys was similar to those pregnant with girls (Table 3). Marked ethnic differences were observed. Compared with NZ European mothers, the incidence rate of antibiotic dispensing was significantly higher among Māori, Pacific, and MELAA mothers, and significantly lower among Asian. Higher dispensing rates were also associated with greater socioeconomic deprivation, urban residence, and younger maternal age.

**TABLE 3:** Median with interquartile range (IQR) of number of antibiotics dispensed during gestation and incidence rate ratios (IRR) comparing different groups.

Explanatory variable	Median (IQR)	Crude IRR (95% CI)	*p*	Adjusted^a^ IRR (95% CI)	*p*
Gender					
Female	0 (0–1)	Ref.	Ref.	Ref.	Ref.
Male	0 (0–1)	1.00 (0.99–1.02)	0.7446	1.00 (0.98–1.01)	0.524
Ethnicity					
NZ Europeans	0 (0–0)	Ref.	Ref.	Ref.	Ref.
Asian	0 (0–0)	0.95 (0.92–0.97)	< 0.0001	0.89 (0.87–0.91)	< 0.0001
MELAA	0 (0–1)	1.23 (1.15–1.31)	< 0.0001	1.16 (1.08–1.23)	< 0.0001
Māori	0 (0–1)	1.59 (1.56–1.61)	< 0.0001	1.30 (1.28–1.32)	< 0.0001
Others	0 (0–0)	0.94 (0.88–1.00)	0.0439	0.95 (0.89–1.01)	0.085
Pacific	1 (0–1)	1.70 (1.67–1.74)	< 0.0001	1.37 (1.34–1.41)	< 0.0001
NZ Deprivation Index in quintiles					
1 (Lowest deprivation)	0 (0–0)	Ref.	Ref.	Ref.	Ref.
2	0 (0–0)	1.10 (1.06–1.13)	< 0.0001	1.06 (1.03–1.09)	< 0.0001
3	0 (0–1)	1.24 (1.21–1.28)	< 0.0001	1.16 (1.13–1.19)	< 0.0001
4	0 (0–1)	1.50 (1.46–1.54)	< 0.0001	1.31 (1.28–1.35)	< 0.0001
5 (Highest deprivation)	0 (0–1)	1.93 (1.188–1.97)	< 0.0001	1.51 (1.48–1.55)	< 0.0001
Rurality					
Rural	0 (0–1)	Ref.	Ref.	Ref.	Ref.
Urban	0 (0–1)	1.05 (1.03–1.07)	< 0.0001	1.08 (1.06–1.10)	< 0.0001
Mode of delivery					
Non‐Caesarean section	0 (0–1)	Ref.	Ref.	Ref.	Ref.
Caesarean section (elective)	0 (0–1)	1.14 (1.12–1.17)	< 0.0001	1.27 (1.24–1.30)	< 0.0001
Caesarean section (emergency)	0 (0–1)	1.06 (1.04–1.08)	< 0.0001	1.09 (1.07–1.11)	< 0.0001
Birth term (in weeks)					
Full‐term (≥ 39 and ≤ 40)	0 (0–1)	Ref.	Ref.	Ref.	Ref.
Pre‐ & early‐term (≤ 38)	0 (0–1)	1.26 (1.24–1.28)	< 0.0001	1.23 (1.21–1.25)	< 0.0001
Late‐term (= 41)	0 (0–1)	0.90 (0.89–0.92)	< 0.0001	0.91 (0.89–0.93)	< 0.0001
Post‐term (≥ 42)	0 (0–1)	0.92 (0.88–0.95)	< 0.0001	0.88 (0.85–0.92)	< 0.0001
Birthweight					
NBW	0 (0–1)	Ref.	Ref.	Ref.	Ref.
HBW	0 (0–1)	0.96 (0.94–0.98)	< 0.0001	1.03 (1.01–1.05)	0.0047
LBW	0 (0–1)	1.38 (1.34–1.41)	< 0.0001	1.15 (1.11–1.18)	< 0.0001
Maternal age at birth					
< 25	0 (0–1)	Ref.	Ref.	Ref.	Ref.
≥ 25 and < 30	0 (0–1)	0.70 (0.69–0.72)	< 0.0001	0.77 (0.75–0.78)	< 0.0001
≥ 30 and < 35	0 (0–0)	0.58 (0.57–0.59)	< 0.0001	0.68 (0.66–0.69)	< 0.0001
≥ 35	0 (0–1)	0.60 (0.59–0.62)	< 0.0001	0.70 (0.68–0.71)	< 0.0001

^a^ Adjusted for sex, ethnicity, deprivation index, rurality, mode of delivery, birth term, birthweight, and maternal age at birth.

## 4 Discussion

This study showed that 96% of New Zealand children aged ≤ 5 years and 30% of pregnant mothers had been dispensed ≥ 1 course of antibiotics. Dispensing was highest among Pacific People and Māori and those from the most deprived neighbourhoods and urban areas. Also, the dispensing rate was higher in certain sub‐groups: those with low and high birthweight, those born via caesarean section, and those born preterm. Mothers of children with low birthweight, those who delivered by caesarean section, and younger mothers also had higher rates of antibiotic dispensing. As expected, dispensing was highest in winter, and penicillin was the most commonly dispensed class of antibiotics.

Another New Zealand study involving 5581 children < 5 years and born between 2009 and 2010 reported a similar level of antibiotic dispensing i.e., 97% [16]. These estimates do not include antibiotics dispensed to children in hospital and the actual antibiotic dispensed is therefore likely higher. Compared to other OECD countries antibiotic consumption is high in New Zealand. For example, in 2013, antimicrobial consumption was shown to be higher than that of 22 (from 29) European countries [30]. Although a subsequent study showed a 4.6% decrease in total population antibiotic dispensing from 2013 to 2018, the rate of decline was notably steeper in children under 5 years [31]. Despite this decline, antibiotic dispensing in New Zealand children in 2018 was still significantly higher compared to countries like the UK, Denmark and the Netherlands i.e., the defined daily doses per 1000 inhabitants per day (DIDs) in 2018 was 22.5 compared to 18.2, 14.0 and 10.1 in the other countries [31]. Also, prescribing may have increased since the introduction of legislation in 2013 permitting non‐physician healthcare professionals to prescribe antibiotics independently [17, 18]. High antibiotic use compared to other countries, combined with higher dispensing frequency in winter when viral respiratory tract infections are more common, points towards overprescription. This practice is of significant concern as it risks a further increase in antibiotic resistance.

Thirty percent of mothers were dispensed antibiotics during pregnancy. The use of antibiotics during pregnancy varies across countries, with rates ranging from 20.8% in the Netherlands to 40.8% in the United States [32]. Urinary tract infections (UTIs) account for most antibiotic use during pregnancy. However, our study was unable to verify specific use as clinical indications are not captured. Again, given the lower rates in some other high‐income countries, it is likely that a significant proportion of antibiotic prescriptions are unnecessary.

We observed a steady increase in antibiotic dispensing during the first 12 months of life. This trend, which is consistent with observations from international studies [33, 34], may be due to several factors, including the high prevalence of respiratory and gastrointestinal infections during infancy and the limited diagnostic tools available for distinguishing between bacterial and viral infections resulting in the precautionary use of antibiotics [35].

During both the prenatal and postnatal periods, we observed a higher proportion of antibiotics dispensed among Pacific, Māori, Asians, and MELAA compared to NZ Europeans. Additionally, these groups tended to receive antibiotics at younger ages. This result is consistent with previous findings in New Zealand [16, 30, 36]. Some of these variations may be explained by differences in treatment need. The risk of serious infectious diseases is known to be markedly higher for Māori and Pacific peoples compared with Europeans and Others [37]. Also, the crude incidence of acute rheumatic fever is 20 times higher in Māori and 44 times higher among Pacific populations than in other ethnic groups in New Zealand [38]. Further, cultural perceptions and trust in healthcare providers may affect antibiotic prescribing [15]. For example, a study by Thaggard, Reid [39] showed that Māori and Pacific peoples' knowledge, perceptions, and expectations regarding antibiotic treatment of URTI's may contribute to higher rates of antibiotic use. This highlights the importance of building community knowledge about antibiotic use alongside the need for safe and effective interventions targeting antibiotic prescribing practices among healthcare professionals.

Our finding that antibiotic dispensing was higher in those with greater deprivation is consistent with previous research conducted by Hobbs, Grant [16] and Norris, Horsburgh [40]. The risk of serious infectious diseases is also markedly higher for people living in more deprived neighbourhoods [37]. Consequently, we would expect to see some association with higher antibiotic prescribing, but probably not to the extent observed. Alternative reasons include poor health literacy leading to carers expecting antibiotics even for viral infections. As noted above, stewardship interventions to mitigate this will need to target parents' expectations as well as those responsible for prescribing antibiotics to ensure appropriate prescribing practices across all socioeconomic groups [22].

Boys were dispensed antibiotics more frequently during the first 2 years of life, with the difference reducing at older ages. The reasons are unclear but may be related to infection susceptibility, societal norms and cultural beliefs, or differences in health‐seeking behaviour between caregivers of male and female infants [41].

Our results showed that children from rural areas were dispensed significantly fewer antibiotics than their urban counterparts. A less pronounced but still statistically significant difference was also observed for mothers, with higher dispensing rates observed for urban mothers. The stronger effect observed in children may be due to differences in healthcare access for acute childhood illnesses. In rural settings, operational constraints such as limited‐service hours can be a barrier, potentially leading to reduced presentations for minor infections. Furthermore, rural healthcare providers may manage these presentations differently, for example by prescribing broad‐spectrum antibiotics or longer courses of therapy, which reduces the need for additional in‐person visits [16, 42]. The reason why this pattern was less pronounced among pregnant mothers could be because a substantial portion of maternal antibiotic dispensing occurs in the context of prenatal cares, which follow standardised national guidelines and may be less influenced by rural vs. urban settings [43].

Children born via C‐section were dispensed more antibiotics, potentially associated with a less well developed immune system due to gut microbiota dysbiosis resulting from reduced exposure to microorganisms at birth, [44] thus potentially increasing the risk of infections in early life [45]. Mothers who delivered via C‐section were also dispensed more antibiotics potentially as part of antibiotic prophylaxis aimed at reducing the risk of postpartum infections and serious complications [46, 47, 48]. Consistent with that, higher antibiotic use was predominantly found during the third and not in the first and second trimesters. We also observed higher antibiotic use among mothers of children with both low and high birthweights. This finding is consistent with previous research reporting an association between antibiotic use during pregnancy and adverse birth outcome, including low birthweight, preterm births and lower Apgar scores [49]. The reason for low birth weight may be explained by prenatal antibiotics altering gene regulation through methylation, which may impact growth, leading to lower birth weight [50]; alternatively, it is plausible that this association is confounded by maternal infections for which the antibiotics were prescribed. It is not clear why prenatal antibiotic dispensing was associated with high birthweight in our study as this finding has not previously been reported.

This study has several strengths, including its reliance on routinely collected data, which is free from non‐response and recall bias. Additionally, given the large size, it allowed for detailed analyses of antibiotic dispensing patterns within specific time frames and across sub‐groups. Also, in contrast to most other studies, we were able to assess antibiotics dispensing of mothers during pregnancy.

There are also limitations to this study. Children who died before age five were excluded, which may introduce selection bias as our findings do not represent this group. However, the proportion of children was very small (< 0.01%), so this is unlikely to have significantly affected the results. Also, pharmaceutical data did not include antibiotics dispensed to children whilst in hospital; antibiotic dispensing reported here is therefore an underestimation, particularly for those who are overrepresented in childhood hospitalisations such as Māori and Pacific children. In addition, antibiotic exposure during pregnancy was restricted to pregnancies resulting in a live birth, which may differ from pregnancies not ending in delivery. Another limitation is the lack of information regarding the clinical indications for which antibiotics were prescribed, thus limiting analysis on the appropriateness of prescribing. Further, we selected children born between 2005 and 2010, and, as discussed, antibiotics prescribing may have changed more recently.

In conclusion, this national cohort showed high antibiotic dispensing, which was highest among Pacific and Māori children, and children in deprived and rural areas. These findings indicate a need for improved and targeted antibiotic stewardship programs, thus reducing the risk of further bacterial resistance in New Zealand and internationally.

## Author Contributions

A.M., J.D., A.K. and M.G.B. conceived the original idea for this data linkage study. All authors contributed to the development of the study methodology. S.R. wrote the first draft of the manuscript, with assistance from J.D., A.M., M.C., A.E., A.K., and M.G.B. All authors, except A.M. (who passed away prior to completing the final draft), read and approved the final manuscript.

## Funding

This work was supported by Health Research Council of New Zealand, 18/509.

## Disclosure

These results are not official statistics. They have been created for research purposes from the Integrated Data Infrastructure (IDI) which is carefully managed by Stats NZ. For more information about the IDI please visit https://www.stats.govt.nz/integrated‐data/.

## Conflicts of Interest

The authors declare no conflicts of interest.

## Supporting information


**Figure S1:** Monthly non‐cumulative and cumulative proportion of infants dispensed antibiotics during the first year of life, among children born between October 2005–December 2010.
**Figure S2:** Percentage of cohort children who were dispensed a course of antibiotic by calendar month and year, restricted to months in which data from the whole cohort were available.
**Figure S3:** Percentage of children who were dispensed one or more antibiotic courses in each year from birth to age 5 years birthweight (S3A_1_ and S3A_2_) and mode of delivery (S3A_1_ and S3B_2_). Figure S3A_1_, B_1_ represent non‐cumulative data and Figure S3A_2_‐S3B_2_ represent cumulative data.
**Figure S4:** Percentage cohort children who were dispensed one or more antibiotic courses in each trimester of life from conception to birth by sex (S4A_1_‐S4A_2_) and rurality (S4B_2_‐S4B_2_). Figure S4A_1_‐S4B_1_ represent non‐cumulative data and Figure S4A_2_‐S4B_2_ represent cumulative data.

## Data Availability

The datasets generated and analysed for this study are not publicly available. Statistics New Zealand's integrated data infrastructure microdata are available for research purposes, and access is granted based on meeting the conditions set by Stats New Zealand through a secure laboratory environment.
