# Supplementary material for: Patterns of Antibiotic Dispensing Among New Zealand Children: A Linkage Study
Source: Pharmacoepidemiol Drug Saf. 2026 Aug 13;35(9):e70450. doi: 10.1002/pds.70450 (PMC13470141; doi:10.1002/pds.70450)
Supplement: Supplementary file 1 [file PDS-35-e70450-s001.docx]

# Supplementary Information

**Figure S1. Monthly non-cumulative and cumulative proportion of infants dispensed antibiotics during the first year of life, among children born between October 2005- December 2010**

**Figure S2. Percentage of cohort children who were dispensed a course of antibiotic by calendar month and year, restricted to months in which data from the whole cohort were available.**

**Figure S3. Percentage of children who were dispensed one or more antibiotic courses in each year from birth to age 5 years birthweight (S3A_1_ and S3A_2_) and mode of delivery (S3A_1_ and S3B_2_). Figures S3A_1_-S3B_1_ represent non-cumulative data and Figures S3A_2_-S3B_2_ represent cumulative data.**

**Figure S4. Percentage cohort children who were dispensed one or more antibiotic courses in each trimester of life from conception to birth by sex (S4A_1_-S4A_2_) and rurality (S4B_2_-S4B_2_). Figures S4A_1_-S4B_1_ represent non-cumulative data and Figures S4A_2_-S4B_2_ represent cumulative data.**

Percent of cohort

| Age (Months) | Non-cummulative | Cummulative |
| --- | --- | --- |
| 0 | 0 | 0 |
| 1 | 2.29739 | 2.24594 |
| 2 | 3.43848 | 5.12742 |
| 3 | 4.60808 | 8.6256 |
| 4 | 6.38005 | 13.0627 |
| 5 | 8.48456 | 18.3194 |
| 6 | 10.6299 | 24.1371 |
| 7 | 12.8247 | 30.3776 |
| 8 | 14.8827 | 36.6954 |
| 9 | 16.3601 | 42.7106 |
| 10 | 17.3758 | 48.1935 |
| 11 | 18.0399 | 53.0462 |
| 12 | 18.3971 | 57.2481 |

**Figure S1. Monthly non-cumulative and cumulative proportion of infants dispensed antibiotics during the first year of life, among children born between October 2005- December 2010**

Percent of cohort

| Time (Month-Year) | Percent |
| --- | --- |
| 2006-01-01 | 2.9 |
| 2006-02-01 | 4.4 |
| 2006-03-01 | 7.7 |
| 2006-04-01 | 7.7 |
| 2006-05-01 | 12.5 |
| 2006-06-01 | 17.9 |
| 2006-07-01 | 21.2 |
| 2006-08-01 | 24.4 |
| 2006-09-01 | 21.5 |
| 2006-10-01 | 18.8 |
| 2006-11-01 | 18.1 |
| 2006-12-01 | 14.6 |
| 2007-01-01 | 10.8 |
| 2007-02-01 | 11.3 |
| 2007-03-01 | 14.3 |
| 2007-04-01 | 13.3 |
| 2007-05-01 | 17 |
| 2007-06-01 | 18.5 |
| 2007-07-01 | 21.6 |
| 2007-08-01 | 23.2 |
| 2007-09-01 | 18.7 |
| 2007-10-01 | 14.5 |
| 2007-11-01 | 13.5 |
| 2007-12-01 | 11.3 |
| 2008-01-01 | 8.1 |
| 2008-02-01 | 9.4 |
| 2008-03-01 | 10.2 |
| 2008-04-01 | 11.3 |
| 2008-05-01 | 12.1 |
| 2008-06-01 | 16.5 |
| 2008-07-01 | 19.7 |
| 2008-08-01 | 19.9 |
| 2008-09-01 | 16.7 |
| 2008-10-01 | 12.1 |
| 2008-11-01 | 10.4 |
| 2008-12-01 | 10.4 |
| 2009-01-01 | 6.8 |
| 2009-02-01 | 9.2 |
| 2009-03-01 | 10.4 |
| 2009-04-01 | 9.6 |
| 2009-05-01 | 11.1 |
| 2009-06-01 | 15 |
| 2009-07-01 | 14.1 |
| 2009-08-01 | 14.4 |
| 2009-09-01 | 13.1 |
| 2009-10-01 | 10.5 |
| 2009-11-01 | 10.7 |
| 2009-12-01 | 9.3 |
| 2010-01-01 | 6.5 |
| 2010-02-01 | 8.7 |
| 2010-03-01 | 10.1 |
| 2010-04-01 | 8.1 |
| 2010-05-01 | 10.9 |
| 2010-06-01 | 12.1 |
| 2010-07-01 | 13 |
| 2010-08-01 | 14.9 |
| 2010-09-01 | 12.5 |
| 2010-10-01 | 6.8 |
| 2010-11-01 | 2.7 |
| 2010-12-01 | 0.6 |

**Figure S2. Percentage of cohort children who were dispensed at least one course of antibiotic by calendar month and year (2005-2010), based on a subset of children born in the last quarter of 2005 (n=8,409).**

| *S3A_1_* | *S3A_2_* |
| --- | --- |
|  |  |
| *S3B_1_* | *S3B_2_* |
|  |  |

**Figure S3. Percentage of children who were dispensed one or more antibiotic courses in each year from birth to age 5 years birthweight (S3A_1_ and S3A_2_) and mode of delivery (S3A_1_ and S3B_2_). Figures S3A_1_-S3B_1_ represent non-cumulative data and Figures S3A_2_-S3B_2_ represent cumulative data. NZ Deprivation Index (in quintiles), DI is 1-least deprived and DI 5 is the most deprived. Birthweights: Low (LBW) = [<2500g]; Normal (NBW) = [2500-4000g]; High (HBW) = [>4000g]. Mode of delivery (birth): ElCS=Elective Caesarean section; EmCS=Emergency Caesarean section; and SVD=Non-Caesarean delivery**

| S4A_1_ | S4A_2_ |
| --- | --- |
| S4B_1_ | S4B_2_ |
|  |  |

**Figure S4. Percentage cohort children who were dispensed one or more antibiotic courses in each trimester of life from conception to birth by sex (S4A_1_-S4A_2_) and rurality (S4B_2_-S4B_2_). Figures S4A_1_-S4B_1_ represent non-cumulative data and Figures S4A_2_-S4B_2_ represent cumulative data.**
